# Targeted deletion of the pancreatic β-cell oxytocin receptor and its effects on metabolic regulation and β-cell health

**DOI:** 10.3389/fendo.2024.1465818

**Published:** 2024-12-23

**Authors:** Armando J. Mendez, Angela Szeto, Maria Boulina, Jesica Westwright, Hafsha Rahman, Sarah Abushamma, Riley Schneider, Philip M. McCabe

**Affiliations:** ^1^ Diabetes Research Institute, Division of Endocrinology, Diabetes and Metabolism, Department of Medicine, University of Miami Miller School of Medicine, Miami, FL, United States; ^2^ Department of Psychology, University of Miami, Coral Gables, FL, United States

**Keywords:** oxytocin receptor, oxytocin, knockout mouse, pancreatic islets, β-cells, insulin, glycemic regulation, cellular protection

## Abstract

The neuropeptide oxytocin (OXT) and its receptor (OXTR) have been shown to play an important role in glucose metabolism, and pancreatic islets express this ligand and receptor. In the current study, OXTR expression was identified in α-, β-, and δ-cells of the pancreatic islet by *in situ* RNA hybridization, and OXT protein expression was observed only in β-cells. In order to examine the contribution of islet OXT/OXTR in glycemic control and islet β-cell heath, we developed a β-cell specific OXTR knock-out (β-KO) mouse. In isolated islets from control mice, OXT enhanced glucose stimulated secretion of insulin, but this response was abolished in the β-KO mice. *In vivo*, supraphysiological doses of OXT reduced blood glucose levels in hyperglycemic Control mice and during a glucose tolerance test. Once again, this response was abolished in the β-KO mice, suggesting that β-cell OXTR may play a role in glycemic regulation. Despite these findings, β-cell deletion of OXTR had no effect on fasting glucose, fasting insulin or glucose tolerance in mice fed a low fat- or high fat-diet for 23 weeks. The low fat or high fat diets did not alter β-cell mass by immundetection or a measure of apoptosis, however, β-KO mice on a high fat diet did exhibit increased β-cell proliferation. In mice treated with the cytotoxic agent, streptozotocin, deletion of OXTR resulted in greater hyperglycemia in β-KO mice relative control mice, suggesting that β-cell OXTR may provide some cytoprotection. In conclusion, the present study provides mixed support for a role of the β-cell OXTR in glycemic regulation. On one hand, *in vitro* experiments and *in vivo* pharmacologic experiments provided evidence that under hyperglycemia, OXTR activation can potentiate insulin secretion and glucose suppression. On the other hand, β-KO followed by chronic dietary manipulation had no effect on whole body glucose regulation *in vivo*. In terms of β-cell health, our data suggests a role of the OXTR in β-cell proliferation and cytoprotection following metabolic or cytotoxic challenge.

## 1 Introduction

The neuropeptide oxytocin (OXT) and its receptor (OXTR) have been shown to play an important role in metabolism. In particular, OXT and OXTR participate in the regulation of appetite via central nervous system (CNS)-neuroendocrine satiety mechanisms (1–4). It has been suggested that hypothalamic OXT neurons act as nutrient status sensors, interacting with cortical and brainstem circuits to decrease food intake (5, 6). In addition to regulating energy intake, OXT and OXTR also play a role in energy expenditure and body composition (5). Deletion of either the OXT or OXTR in mice leads to the development of late-onset obesity without any alterations in food intake (7–11). Exogenous OXT adminstration leads to weight loss above and beyond what would be expected based on its impact on food intake alone, suggesting that OXT/OXTR may increase energy expenditure (12, 13). Consistent with this notion, OXT/OXTR can promote lipolysis and lead to decreases in body fat mass, while preserving or increasing lean mass (14–16). Another reported metabolic effect of OXT is that high-fat fed animals treated with OXT show improvements in glucose tolerance, suggesting changes in insulin sensitivity (13). Consistent with this notion, deletion of the OXT gene leads to decreased insulin sensitivity and glucose intolerance (7). Interestingly, OXT has been shown to induce secretion of both insulin and glucagon from the pancreas (17–19).

It has been suggested that endoplasmic reticulum (ER) stress and inflammation play a role in β-cell dysfunction and diabetes (20, 21). Interestingly, it has been shown that a physiological dose of OXT attenuated cell death induced by metabolic stressors (cytokines, palmitate, and tunicamycin) in isolated mouse pancreatic islets (22). It was further demonstrated that in islets of OXTR^-/-^ mice, metabolic stress increased expression of genes implicated in ER stress and impaired signaling pathways involved in the ER stress response. In another study, it was shown that OXT was protective against streptazocin- and cytokine-induced β-cell death, and OXT enhanced proliferation of cultured human and rat β-cells (23). A subsequent study reported that the administration of oxytocin peptide-analogs in mice reduced β-cell mass and apoptosis following a high fat diet (23). Taken together these studies provide evidence that OXT/OXTR may play a role in beta cell protection in response metabolic stressors.

The peripheral effects of OXT are classically thought to result from hypothalamic-posterior pituitary release into the systemic circulation, yet both OXT and OXTR have been detected in peripheral tissues, including the endocrine pancreas. Using a rat model, OXTR mRNA was reported to be localized in both pancreatic α- and β-cells by immunohistochemistry, with α-cells showing greater immunoreactivity (24). Another study found OXTR mRNA in mouse islets and in a β-cell line (MIN6), but minimal expression of OXT (22). In contrast, Mohan et al. (23) reported significant OXT gene expression in mouse islets, as well as OXTR expression in rat islets, the rat BRIN BD11 β-cell line, and the human 1.1B4 β-cell line. In the mouse islets, there was co-localization of OXT with both insulin and glucagon, with β-cells showing significantly stronger OXT labeling than α-cells. Finally, using the green fluorescent protein Venus under the control of the OXT promoter, it was reported that Venus and OXT immunosignals were colocalized with insulin immunoreactive cells in rat pancreatic islets (25). Taken together, these findings raise the possibility that the metabolic and cell-protective effects of OXT in the endocrine pancreas may not only be due to CNS/pituitary-derived OXT but could also result from a local pancreatic OXT/OXTR network.

Although it has been established that OXT and OXTR play a role in metabolism, the relative contributions of central versus peripheral OXT/OXTR are not clear. In particular, the role of the pancreatic OXT/OXTR system in metabolic regulation and β-cell protection is not well understood. Therefore, we developed a β-cell specific OXTR knock-out (β-KO) mouse to study how this receptor pathway influences β-cell function and survival under conditions of metabolic or inflammatory stress. Through the use of this targeted β-cell OXTR deletion, we examined insulin secretion in isolated islets from β-KO mice, and glycemic regulation under conditions of euglycemia versus insulin resistance induced by a high fat diet (HFD). We also evaluated the role of β-cell OXTR on β-cell protection and proliferation in HFD mice. It was hypothesized that selective OXTR deletion from β-cells would impair insulin secretion during metabolic challenge, leading to impaired glycemic regulation. It was also hypothesized that β-cell OXTR knockout mice would exhibit decreased β-cell mass and increased β-cell proliferation following metabolic or inflammatory stress. Finally, in response to a cytotoxic challenge, it was hypothesized that OXTR deletion would accelerate β-cell dysfunction.

## 2 Materials and methods

Reagent manufacturer and catalog numbers are provided in **Supplementary Materials**.

### 2.1 Experimental animals and generation of β-KO mice

All animal studies were reviewed and all procedures were approved by the Institutional Animal Care and Use Committee of the University of Miami, and conformed to the Guide for the Care and Use of Laboratory Animals published by the US National Institutes of Health (26).

Mice heterozygous for the Oxtr^flox^ targeted allele flanking exons 2-3 of the OXTR gene (B6.129(SJL)-Oxtrtm1.1Wsy/J; Stock: 008471) and Ins1^cre^ mice expressing Cre recombinase (B6(Cg)-Ins1tm1.1(cre)Thor/J; Stock: 026801) and C57BL/6J (Stock: 000664) mice were obtained from the Jackson Laboratory (Bar Harbor, ME, USA).

To develop mice with β-cells-specific deletion of OXTR, the Oxtr^flox^ heterozygous mice were crossed with each other to generate the homozygous genotype. These mice were mated with the homozygous Ins1^cre^ mice expressing Cre recombinase (27, 28). Breeding the two homozygous lines yielded F1 heterozygous mice containing one copy of each gene. The F2 offspring were genotyped (Transnetyx, Cordova, TN) shortly after weaning and housed in same-sex groups, separated by genotype. Males and females of correct homozygous genotype were then bred to produce homozygous litters (F3).

Although it is important to examine gender differences in metabolic studies, it has been shown that both OXT and OXTR expressions vary significantly during the estrus cycle (29). Therefore, to simplify the current study, we chose to focus on male mice.

### 2.2 *In vivo* metabolic studies

Experiment 1. At 9 weeks of age, OXTR β-cell KO (β- KO) and Oxtr^flox^ control mice were randomized into two groups to receive either a standard rodent chow low-fat diet (LFD; Inotiv, Inc., Indianapolis, IN) or a high-fat diet (HFD; 60 Kcal% from fat; Research Diets, New Brunswick, NJ). After randomization, mice were acclimated (4 to 5 animals/cage) for one week after which the HFD was started. Mice were housed in animal facilities where they were given ad libitum food and water on a 12:12 light dark cycle.

Experiment 2. In a second cohort of 21-week-old mice control and KO, randomized and housed as above, were fed HFD and followed for 40 weeks.

All other experiments were performed on mice maintained on a LFD under conditions described in the Figure Legends.

### 2.3 Metabolic testing

An intraperitoneal (i.p.) glucose tolerance test (ipGTT) was performed after a 6 hour or overnight (16 to 18h) fast Animals received a bolus i.p injection of glucose (2 g/kg) in saline. Blood glucose was measured from the tail vein at baseline, 15, 30, 60, 90 and 120 min after glucose administration. In some cases, approximately 50 µl of blood was collected by mandibular blood draw at baseline for hormone measurements. Insulin tolerance tests (0.8 U/kg) were performed after removing food from the cages for 1 hour and blood glucose measured over 90 minutes. OXT was administered by i.p injection at the indicated concentrations after a 4 fast or just prior to an ipGTT. Blood glucose was measured after 15, 30, 45, 60, 90, and 120 min. Blood glucose was measured with a glucometer (Contour Next, Bayer, Mishawaka, IN). The area under the curve calculations used the trapezoidal rule calculated with GraphPad Prism software (Boston, MA).

### 2.4 Necropsy

Mice were sacrificed by CO_2_ asphyxiation at the end of each study. Blood was collected by cardiac puncture, transferred to a 1 ml Microvette tube containing EDTA (Sarstedt, Newton, NC, USA) and placed on ice until separation of plasma by centrifugation. Organs were weighed and the tissues were either snap-frozen in liquid nitrogen and stored at −80°C or fixed in 10% neutral buffered saline.

### 2.5 Streptozotocin treatment

Male and female β- KO and control mice between 18 and 20 weeks of age were maintained on a LFD and given low-dose STZ (i.p.; 50 mg/kg in 0.1 M citrate buffer, pH 4.5; Cayman Chemical, Ann Arbor, MI) for 4 consecutive days. Mice were fasted for 4 hours prior to STZ injection (30). Body weight and non-fasting blood glucose were monitored over 21 days.

### 2.6 Static glucose stimulated hormone secretion by isolated islets

Islets from β- KO and control mice were isolated using collagenase digestion as previously described (31). Isolated islets were cultured in RPMI 1640 supplemented with 10% fetal bovine serum, 100 U/ml penicillin, and 0.1 mg/ml streptomycin for 24 h at 37°C and 5% CO_2_ prior to use in experiments. After isolation, islet media was replaced by washing with Kreb’s buffer saline (KBS; 114 mM NaCl, 4.72 mM KCl, 26 mM NaHCO_3_, 1.2 mM KH_2_PO_4_, 1.2 mM MgCl_2_·7H_2_O, 2.56 mM CaCl_2_·2H_2_O, 25 mM HEPES, 0.1% bovine serum albumen (BSA), pH 7.4). Pooled islets were isolated from the pancreata of ten control or ten KO mice. Approximately 50 islets were placed into 3 µm trans-well inserts (Millipore, Burlington, MA) placed in a 24-well plate, equilibrated in Krebs buffered saline (KBS) containing 0.1% BSA and 1 mM glucose for 1 hour then sequentially incubated in the same buffer containing 3mM glucose for 1 hour followed by 1 hour with 16.7 mM glucose and with or without 1nM oxytocin. After each incubation the buffer was collected for measurement of insulin and glucagon. After incubation the islets in the trans wells were washed 4 x with PBS and the islets were then solubilized in T-PER extraction buffer (Thermo Fisher, Waltham, MA) for determination of total protein. Media samples were stored frozen until used for assay.

### 2.7 Insulin and glucagon immunoassays

Insulin and glucagon from β-KO and wild type mice and the media collected from glucose-stimulated insulin secretion from isolated islets was measured for insulin and glucagon. Insulin and glucagon were measured by ELISA (Mercodia, Winston-Salem, NC).

### 2.8 RT-qPCR gene expression

Real time quantitative RT-PCR was used to quantify tissue OXTR mRNA expression levels. Total RNA was isolated using RNeasy extraction kit according to manufacturer’s protocol (Qiagen, Valencia, CA, USA), then cDNA was synthesized after DNase I treatment using reagents from Applied Biosystems (Foster City, CA, USA) following manufacturer’s instructions. Quantitative expression of genes by RT-qPCR was performed with the TaqMan gene expression assay. The following Applied Biosystems inventoried primers were used: mouse OXTR, INS2, AVPR1a, AVPR1b, GCG, PDX1, and OXT. cDNA (50 ng) was amplified with TaqMan Universal PCR Master Mix and reactions were run using universal cycling conditions on an Applied Biosystems 7500 Real-Time qPCR system. Samples were analyzed in triplicate. The ΔΔCT threshold cycle method was used to analyze changes in gene expression. Relative quantification (RQ) was expressed as the fold change compared to the appropriate control condition (32). 18S rRNA was used as the endogenous RNA control. A non-template control was performed to ensure that there was no amplification of genomic DNA.

### 2.9 Immunohistochemistry

Formalin-fixed paraffin-embedded (FFPE) pancreas tissue was sectioned at 8 µm. Sections were deparaffinized and antigen retrieval was performed by heating at 100°C in a decloaking chamber for 10 minutes in citrate buffer (pH = 6.0). Slides were washed with phosphate-buffered saline (PBS), incubated with blocking solution (PBS containing 1% bovine serum albumin and 10% donkey serum) then incubated with antibodies. The primary antibodies were guinea pig anti-insulin (Dako, Santa Clara, CA), mouse anti-glucagon for tissues (Santa Cruz Biotechnology, Dallas, TX), oxytocin (Proteintech, Rosemont, IL), and somatostatin (Thermo Fisher, Waltham, MA). Antibodies were diluted in blocking buffer and incubated overnight at 4°C. Nonimmune immunoglobulins from the same species were used to assess nonspecific staining. Slides were rinsed with wash buffer (Biogenex, Fremont, CA) and then incubated with secondary antibodies: donkey anti-guinea pig 488, donkey anti-rat 594 or donkey anti-mouse 594, donkey anti-mouse 647, donkey anti-rabbit 649 and 790 (Jackson ImmunoResearch, West Grove, PA). Secondary antibodies were incubated for 2 hours in the dark at room temperature followed by rinsing with wash buffer and then PBS. Finally, slides were mounted using ProLong Gold (Life Technologies, Grand Island, NY).

For β-cell mass estimation, 5 sections separated by 100 µm were used for insulin staining. An equal number of slides from each group was analyzed in batches. β-cell area was estimated by insulin positive immunoreactive area expressed as a percent of the total pancreas area. The whole tissue area was measured based on DAPI staining.

β-cell apoptosis was analyzed by terminal deoxynucleotidyl transferase-mediated dUTP nick-end labeling (TUNEL) staining according to the manufacturer’s instructions (Roche Life Sciences, Indianapolis, IN). Apoptotic cells in pancreatic islets were expressed as the number of TUNEL-positive cells per area of pancreatic islets (cells/mm^2^). Quantification of β-cell proliferation was determined by expression of Ki67 (Thermo Fisher, Waltham, MA). Ki-67 immunoreactive cell nuclei were counted in the same tissue sections and normalized for total β-cell immunoreactive area.

### 2.10 *In situ* mRNA hybridization

*In situ* hybridization was performed according to the manufacturer’s instructions for FFPE tissue using the RNAscope Multiplex Fluorescent Reagent Kit v2 (Advanced Cell Diagnostics, Newark, CA). Briefly, fixed pancreas sections (8 µm) were heated at 37°C in an oven for at 60 min, deparaffinized then slides were heated for 15 min at 60°C. Sections were treated with hydrogen peroxide for 10 min at room temperature followed by antigen retrieval (100°C for 15 minutes in citrate buffer, pH = 6.0). Sections were treated with Protease Plus, placed into the HybEZ Humidity Control tray (Advanced Cell Diagnostics, Newark, CA) and placed in the HybEZ oven (Advanced Cell Diagnostics, Newark, CA) for 30 min at 37°C and then rinsed 3 x 5 min with distilled water prior to being incubated with the target probes in the oven for 2h at 40°C. To evaluate the OXTR expression in control and KO animals, a custom probe that spanned the region removed by the Cre recombinase in the OXTR gene (27) designed by Advanced Cell Diagnostics and is now commercially available. The probes were: custom OXTR, insulin, glucagon, and somatostatin. Following the 2h-incubation and amplification, sections were washed then incubated with the fluorescence labelled probe for 30 min at 40°C (Opal 570 nm), green (Opal 520 nm) and far red (Opal 650 nm; Perkin Elmer Reagent Kit, 4-color kit) and mounted in ProLong Gold.

### 2.11 Microscopy and Image analyses

Images of entire pancreatic sections were acquired using an Olympus immunofluorescence slide scanner microscope (VS120, Center Valley, PA; VS120 Slide Scanner housed at the University of Miami Miller School of Medicine Analytical Imaging Core Facility) with a 20X objective lens that resulted in a 640-nm/pixel resolution. High-resolution confocal images were acquired using a Leica SP5 inverted confocal microscope with a 20X/0.70 NA objective lens. Adjusted images were then used to quantify the immunoreactive areas with FIJI Image J (33, 34) using color thresholding with the default setting, saturation and brightness settings to select the color positive immunoreactive regions for all images. β-cell mass was measured using batch processing with FIJI software. The images were adjusted between batches to accommodate for variation between batches. A minimum of 5 individual whole-pancreatic slides (for each sample) were imaged and analyzed.

### 2.12 Statistical analyses

All *in vitro* experiments were reproduced at least three times. The *in vivo* experiments were performed with at least 6-8 animals per group and no mice were excluded from the statistical analysis. Data were shown to be normally distributed using the Shapiro-Wilk test and outliers were identified using the ROUT analysis in GraphPad Prism 10. Data are presented as means ± standard error of the mean (SEM). Results were compared by unpaired Student’s t-tests or ANOVA (one or two-way) with *post hoc* Bonferroni correction, or the Mann-Whitney U nonparametric test for data that was not normally distributed. An α-level of 0.05 was required for statistical significance.

## 3 Results

### 3.1 OXT and OXTR expression in pancreatic islets

**Figure 1:**
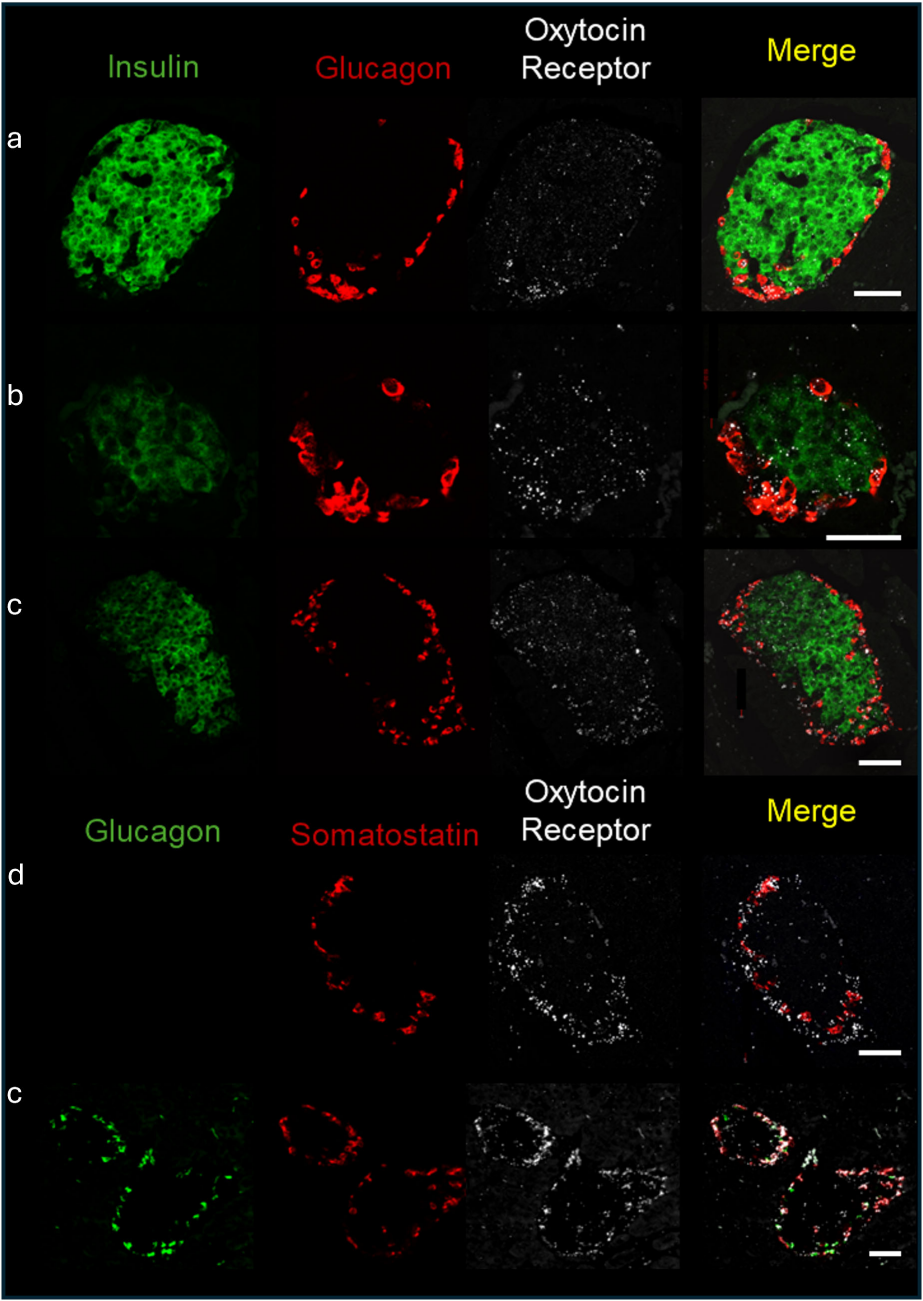
Expression of the oxytocin receptor within the islets of Langerhans of C57BL/6J mice. *In situ* hybridization of the oxytocin receptor, insulin, glucagon, and somatostatin was performed in formalin fixed pancreatic tissue sections and representative images of islets from 4 different C57BL/6J mice (identified by letters (a-d) are shown. Scale bar = 50µm.

**Figure 2:**
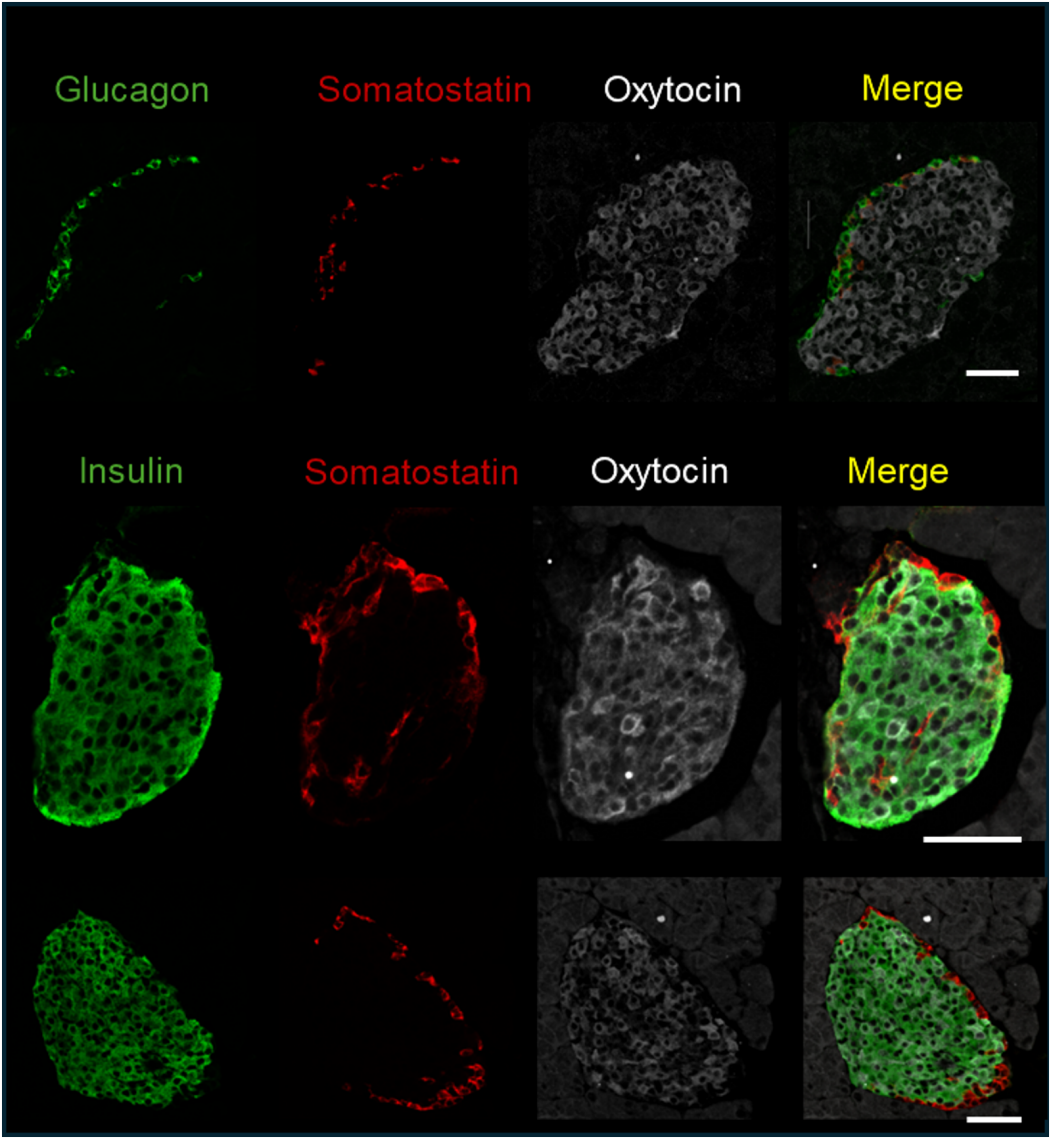
Immunofluorescence detection of insulin, glucagon, somatostatin, and oxytocin in fixed pancreatic tissue; representative images of islets from 3 C57BL/6J mice are shown. Scale bar = 50µm.

As mentioned in the introduction, OXTR and OXT expression have been reported in pancreatic islets. To determine which cell types expressed OXTR in control mouse islets, pancreatic tissue sections were used for *in situ* hybridization to detect OXTR, glucagon (α-cells), somatostatin (δ-cells) and insulin (β-cells) mRNA (**Figure 1**). There was strong expression of OXTR in α-and δ-cells. Notably, expression in β-cells was sparse and generally the signal was less intense than in alpha and delta cells, and not all β-cells exhibited an OXTR signal. We also evaluated the presence of OXT in islet cells by immunofluorescence (**Figure 2**). Images reveal that immunoreactive OXT was only detected in β-cells and was not seen in alpha or delta cells.

### 3.2 β-cell specific deletion of the OXTR gene

To study the role of the OXTR/OXT system specifically in insulin-producing cells, we established a β-cell-specific OXTR-KO mouse model by breeding mice expressing a floxed OXTR gene with mice expressing Cre recombinase under the control of the insulin promoter to obtain pancreatic β-KO mice. The genotype of these mice, characterized by the loss of the floxed sequence and presence of the CRE construct, was confirmed as described in the Methods Section. This tissue-specific KO can provide information on the role of the OXT/OXTR system specific to the β-cell, without perturbing other OXT target tissues.

**Figure 3:**
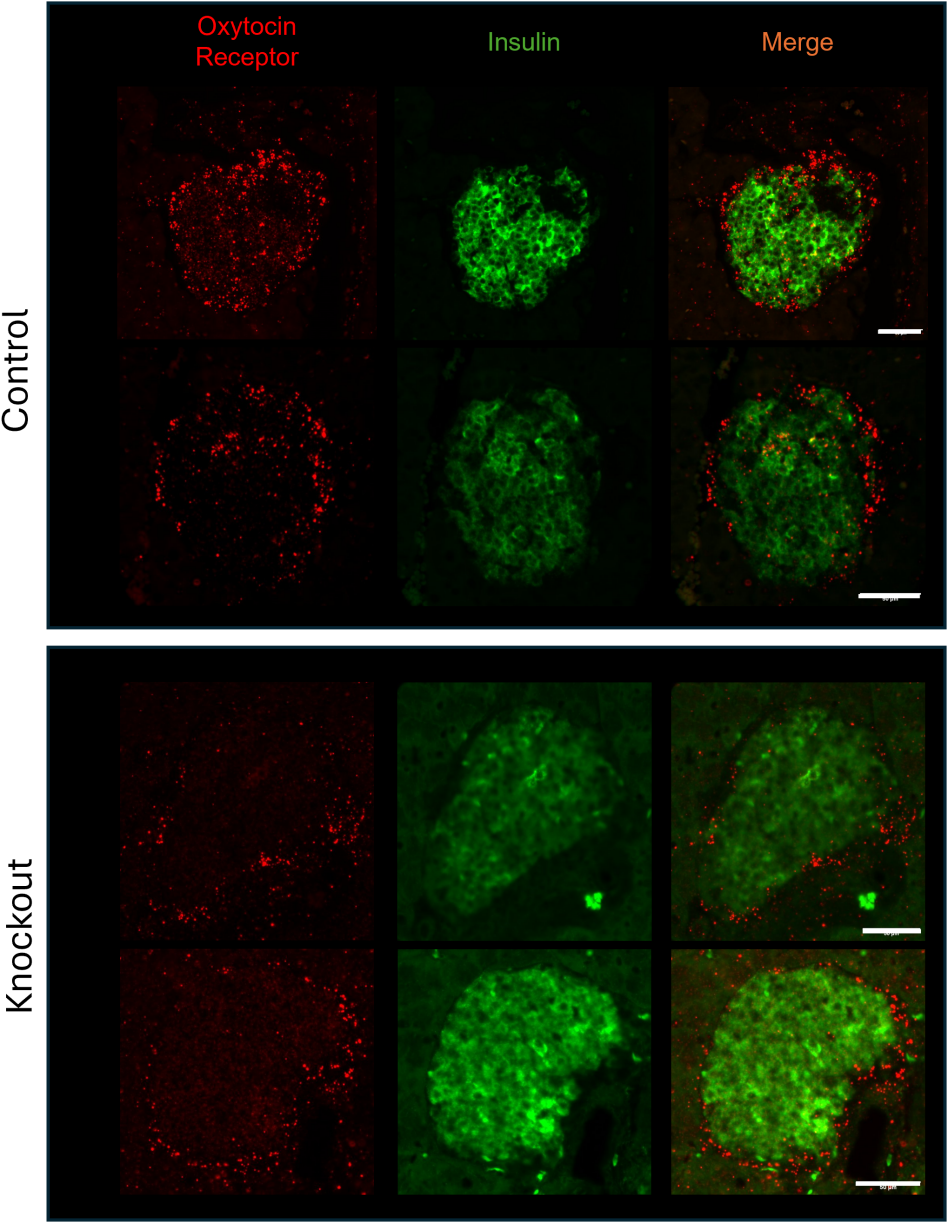
Expression of the oxytocin receptor and insulin in islets from wild type and β-cell OXTR knockout mice detected by *in situ* hybridization. Images are from representative islets from 2 control and 2 knockout mice. Scale bar = 50µm.

To confirm that β-KO mice had reduced OXTR expression, we compared the levels of OXTR mRNA in isolated mouse islets from control and β-KO mice (n= 3 islet isolations per group, each isolation represented islets pooled from 5 male mice). Expression of OXTR mRNA was decreased by 39.7 ± 8% (p = 0.009) in OXTR-KO islets compared to the controls. This result is consistent with the deletion of OXTR from β-cells, but not from α-and δ-cells, and suggests that α-and δ-cells which together comprise <30% of the islet cells (35), account for 60% of OXTR expression in the islet. In addition, there were no significant differences in the mRNA expression of INS2, GCG, PDX1, OXT, or the vasopressin receptors AVPR1a and AVPR1b between control and β-KO islets (**Supplementary Table 1**). To confirm specificity of the knockout to β-cells, we compared levels of OXTR mRNA in brain, heart, and epididymal fat tissues and expression was not different between control and β-KO mice (**Supplementary Table 2**). To identify the cells that had reduced OXTR mRNA expression, we again used *in situ* RNA hybridization with pancreatic tissues from control and β-cell KO mice (**Figure 3**). In control mice, OXTR expression could be observed in regions that colocalize with insulin mRNA as well as in cells in the periphery of the islet consistent with α-and/or δ-cell expression. In contrast, the β- KO mice had essentially no OXTR expression within insulin positive cells while retaining expression in non-insulin expressing cells. Taken together, these data confirm that OXTR expression was undetectable in the β-cells of KO mice, and that approximately 40% of the total islet OXTR expression in control mice is present in β-cells.

### 3.3 Functional Validation of the β-KO in isolated islets

**Figure 4:**
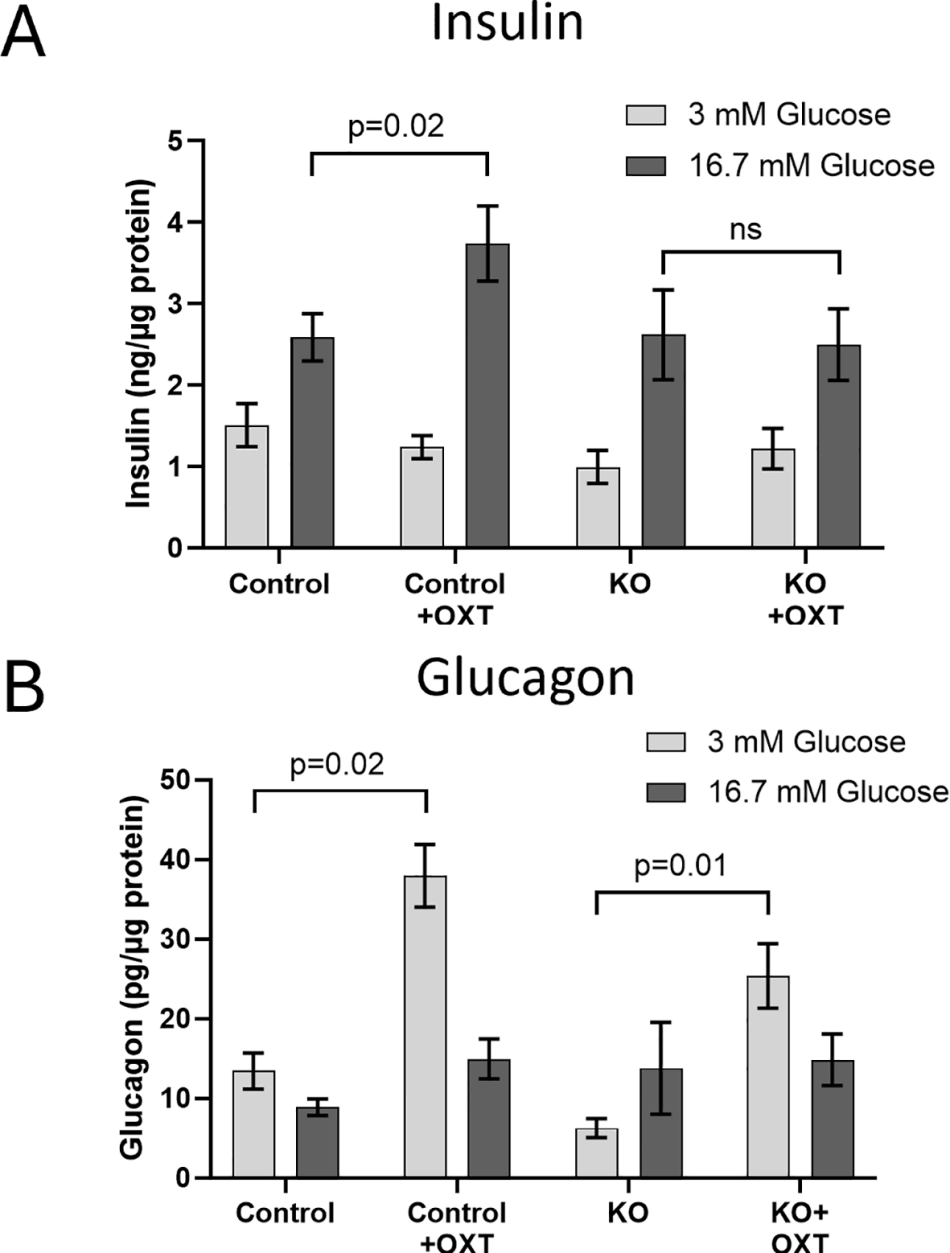
Effects of oxytocin on glucose-stimulated insulin **(A)** and glucagon **(B)** secretion in isolated islets from control and β-cell OXTR knockout (KO) mice. Approximately 50 islets were placed into trans-well plates, equilibrated in Krebs buffered saline (KBS) containing 0.1% BSA and 1 mM glucose for 1 hour then sequentially incubated in the same buffer containing 3mM glucose for 1 hour followed by 1 hour with 16.7 mM glucose and with or without 1nM oxytocin. After each incubation the buffer was collected for measurement of insulin and glucagon. Hormone secretion was expressed as ng of insulin or pg of glucagon secreted per µg of islet protein. Data are the mean ± SEM of four wells per incubation and p values were determined by one-way ANOVA. ns indicates not significant.

To show that the β-KO resulted in a functional loss of OXTR activity, we evaluated the ability of exogenous OXT to stimulate insulin and glucagon secretion from isolated islets from control or β-KO mice (**Figure 4**). As expected, insulin secretion from control islets increased when exposed to high glucose (16.7 mM) relative to low glucose (3mM) following sequential incubation of the same islets. When 1 nM OXT was included during the incubations, there was no effect on basal stimulation at low glucose, however, in the presence of high glucose, oxytocin potentiated insulin secretion (**Figure 4A**). In islets from β-KO mice, insulin secretion was similar to control islets under low and high glucose incubations, however, OXT had no effect on insulin secretion in the islets from the β-KO mice under any condition. Glucagon secretion in control islets was not significantly different in low or high glucose conditions, however in the presence of OXT, glucagon secretion was significantly increased by OXT under low glucose, but not high glucose compared to controls. A similar pattern of glucagon secretion and stimulation by OXT in low glucose conditions was also observed in islets from the β-KO mice (**Figure 4B**). These data demonstrate that OXT, working through its receptor, produces an incretin-like response in insulin secretion from isolated islets, which is abolished by the β-KO.

### 3.4 OXT/OXTR modulation of glycemic control *in vivo*

**Figure 5:**
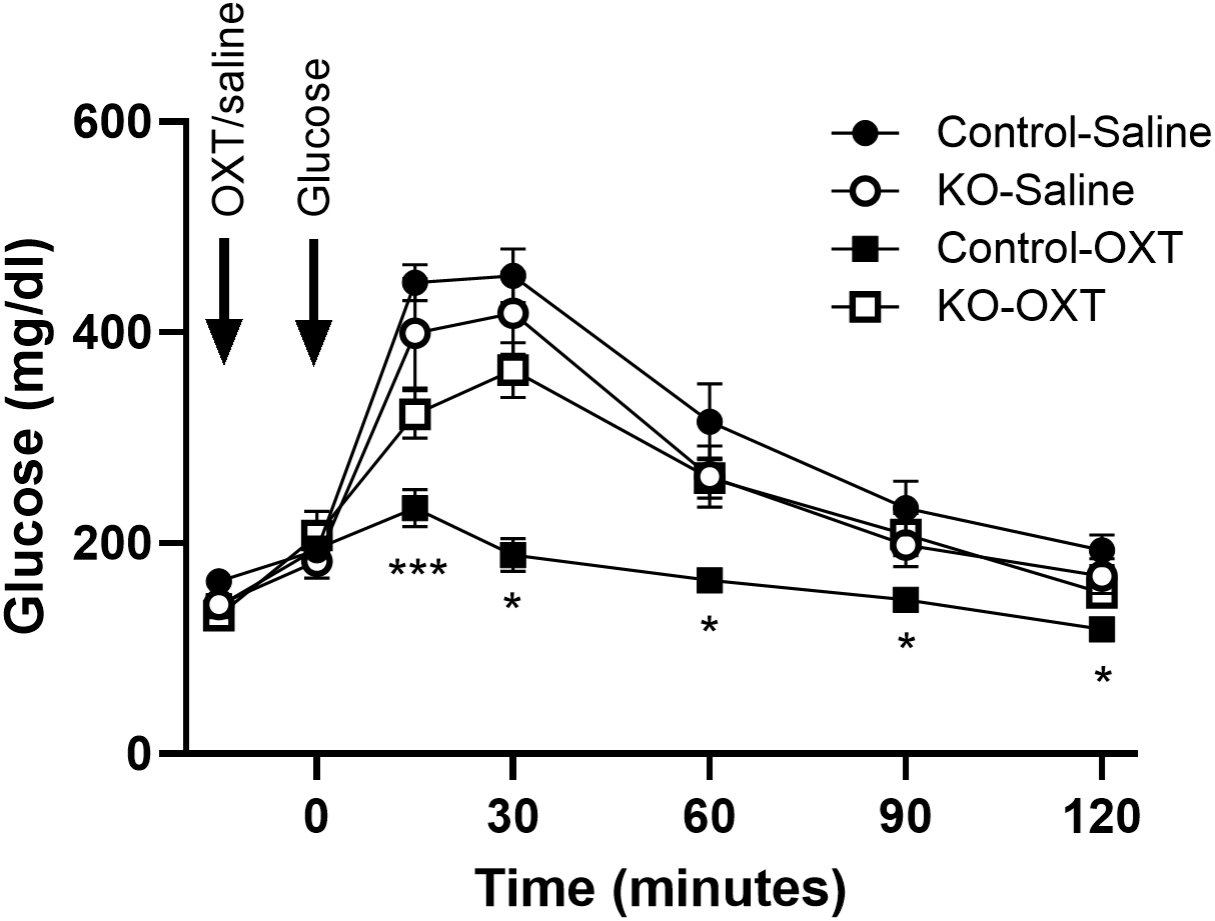
Effect of exogenous oxytocin (OXT) administration on the ipGTT in control and β-cell OXTR knockout (KO) mice. Male mice (27 weeks of age) maintained on a LFD diet were fasted for 6 hours then received saline or 1 mg/kg OXT i.p injections followed by an i.p. injection of glucose 10 minutes later. The glucose excursion was evaluated over 2 hours, n= 7 or 8 per group from 2 experiments. Data are expressed as the mean ± SEM; *, *** indicates p < 0.05 or <0.001, respectively.

We evaluated the effects of oxytocin administration to mice during an ipGTT and show the OXT dose-response on the glucose excursion and the glucose area under the curve in control mice (**Supplementary Figures 1A, B**). Increasing doses of OXT progressively decreased the blood glucose during the test, with significant effects at 1 µg/kg body weight and maximal effects observed at 100 and 1000 µg/kg. We subsequently tested whether the decrease in glucose would be observed under normal glycemic or hyperglycemic states. control lean mice were fasted for 4 hours, injected with OXT (1000 µg/kg) or saline, and then blood glucose was monitored over 2 hours. As shown in **Supplementary Figure 1C**, there were no significant differences in blood glucose at any time point in the mice treated with OXT compared to the saline control group. In mice made hyperglycemic by a HFD, injection of OXT resulted in a significant decrease in blood glucose compared to the control group (**Supplementary Figure 1D**). These data demonstrate that the ability of OXT to reduce blood glucose is context dependent in that the effect was only observed when blood glucose was elevated. Finally, to evaluate whether this effect of OXT persisted after OXTR knockout, blood glucose levels during an ipGTT were evaluated in the control and β-KO mice (**Figure 5**). In mice given saline, there were no differences in the glucose excursion over time between the control and β-KO mice. In control mice that received OXT, the glucose excursion was significantly blunted compared to the saline control group (p=0.032). However, in the β- KO mice, OXT administration was without significant effect relative to the saline control group. These data demonstrated that the ability of oxytocin to prevent the glucose excursion during the GTT is specifically lost in the β-KO mice, further validating the KO phenotype under *in vivo* conditions. It should also be noted that these effects were only seen at supraphysiological OXT levels (see Discussion).

**Figure 6:**
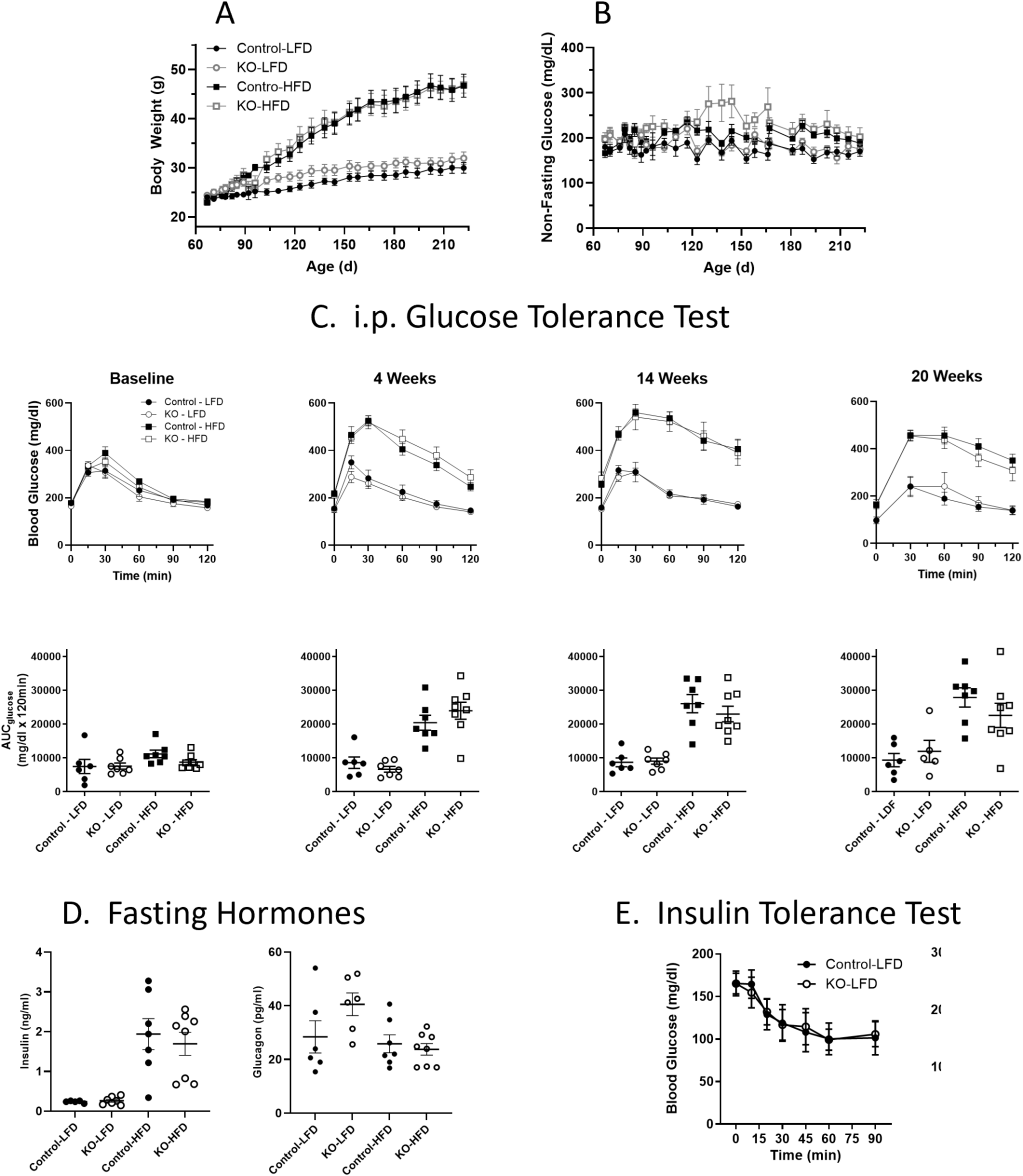
Body weight (Panel **A**) and non-fasting blood glucose levels **(B)** in control and β-cell OXTR knockout (KO) mice were maintained on a low-fat diet (LFD) or high-fat diet (HFD) over a 23-week study period. Data are the mean ± SEM, Control-LFD n=6, KO-LFD n=7, Control-HFD n=7 and KO-HFD n=8. **(C)** Mice were fasted for 6 hours, and glucose tolerance tests were performed throughout the study on Control and KO mice fed a LFD or HFD, n= 7 or 8 per group. The area under the curve for glucose (AUC_glucose_) from the GTT data is shown below. **(D)** Insulin and glucagon levels in control and β-cell OXTR knockout (KO) mice fasted for 16 hours at study week 20. **(E)** Insulin tolerance test was performed on control and β-cell OXTR knockout (KO) mice on the LFD or HFD. Mice were fasted for 4 hours prior to injection of insulin and blood glucose levels were monitored over 2 hours. There were no significant differences between control and KO within each diet group.

Studies were performed to evaluate whether glucose homeostasis and/or insulin sensitivity was affected *in vivo* in β-KO relative to the control mice. Mice were maintained on either a LFD or on a HFD starting at 8 weeks of age and monitored for 23 weeks. Animals receiving the HFD showed increased weight relative to LFD mice (**Figure 6A**), but there were no significant differences between control and β-KO mice in either dietary group. In addition, non-fasting glucose was monitored in all animals and no significant differences were observed between any group (**Figure 6B**). As assessed by an ipGTT (**Figure 6C**) HFD animals became hyperglycemic (evidenced by higher fasting glucose at baseline compared to the LFD mice) and had impaired glucose tolerance by 4 weeks, that progressed over the course of the study (as seen by the increased glucose excursion and increased glucose area under the curve). Despite the impaired glucose regulation in the HFD mice, there was no difference in the glucose tolerance profile between the β-KO and control mice. Similarly, there were no group differences in ipGTT in LFD mice. Further, no differences in fasting insulin or glucagon levels between control and β-KO mice on either diet were observed (**Figure 6D**). We also evaluated insulin sensitivity after an i.p. insulin injection in mice at week 18 of the study (**Figure 6E**). Mice in the HFD group showed impaired insulin tolerance compared to the LFD group, but there were no differences between β-KO and control mice for either dietary condition. These data suggest β-cell OXTR does not play a major role in glycemic regulation under these experimental conditions.

**Figure 7:**
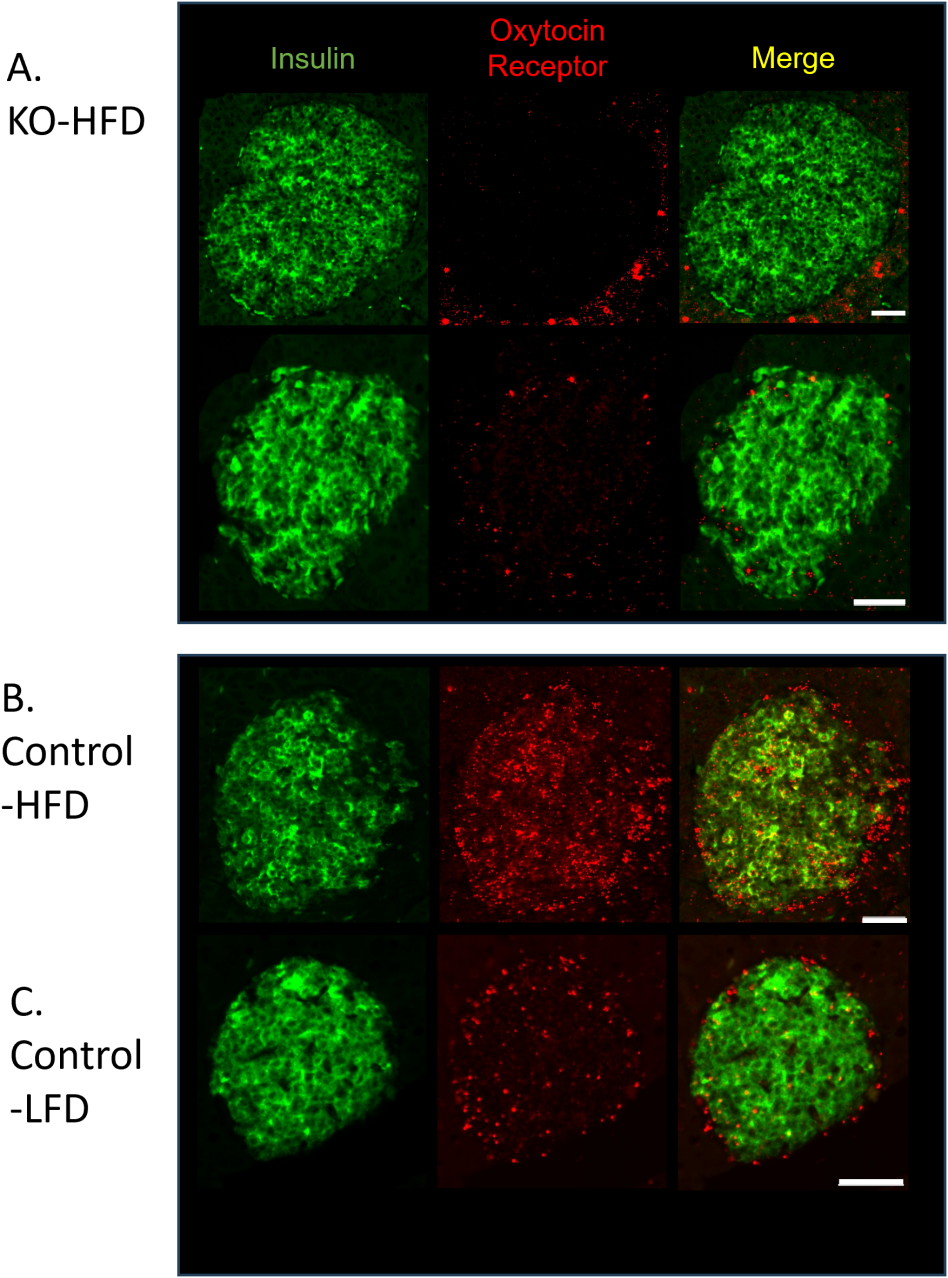
Expression of insulin and the oxytocin receptor in islets from β-cell OXTR knockout (KO; **A**) and control **(B)** mice detected by *in situ* RNA hybridization. Representative images are from mice maintained on a high-fat diet (HFD) or low-fat diet (LFD). **(C)** Scale bar = 50µm.

To establish that the β-KO deletion persisted over the course of the dietary manipulation, tissues from the study cohort were analyzed by *in situ* hybridization for the presence of OXTR mRNA in β-cells. In the β-KO-HFD mice there was no detectable OXTR signal in β-cells (**Figure 7A**) or in β-KO-LFD mice (e.g., **Figure 3**), thereby confirming the OXTR deletion. However, it was noted that the OXTR mRNA signal appeared to be upregulated in the control-HFD mice (**Figure 7B**) compared to the control-LFD mice (**Figure 7C**). Based on the integrated fluorescence density (IFD) of the OXTR in β-cells, control mice on the HFD exhibited a ~5-fold increase in OXTR expression compared to mice on the LFD (**Figures 7B, C**; 2798 ± 531 vs 535 ± 165 IFD/insulin positive area, respectively, p = 0.015, n= 16 to 40 islets per pancreas from 3 pancreata per group).

**Figure 8:**
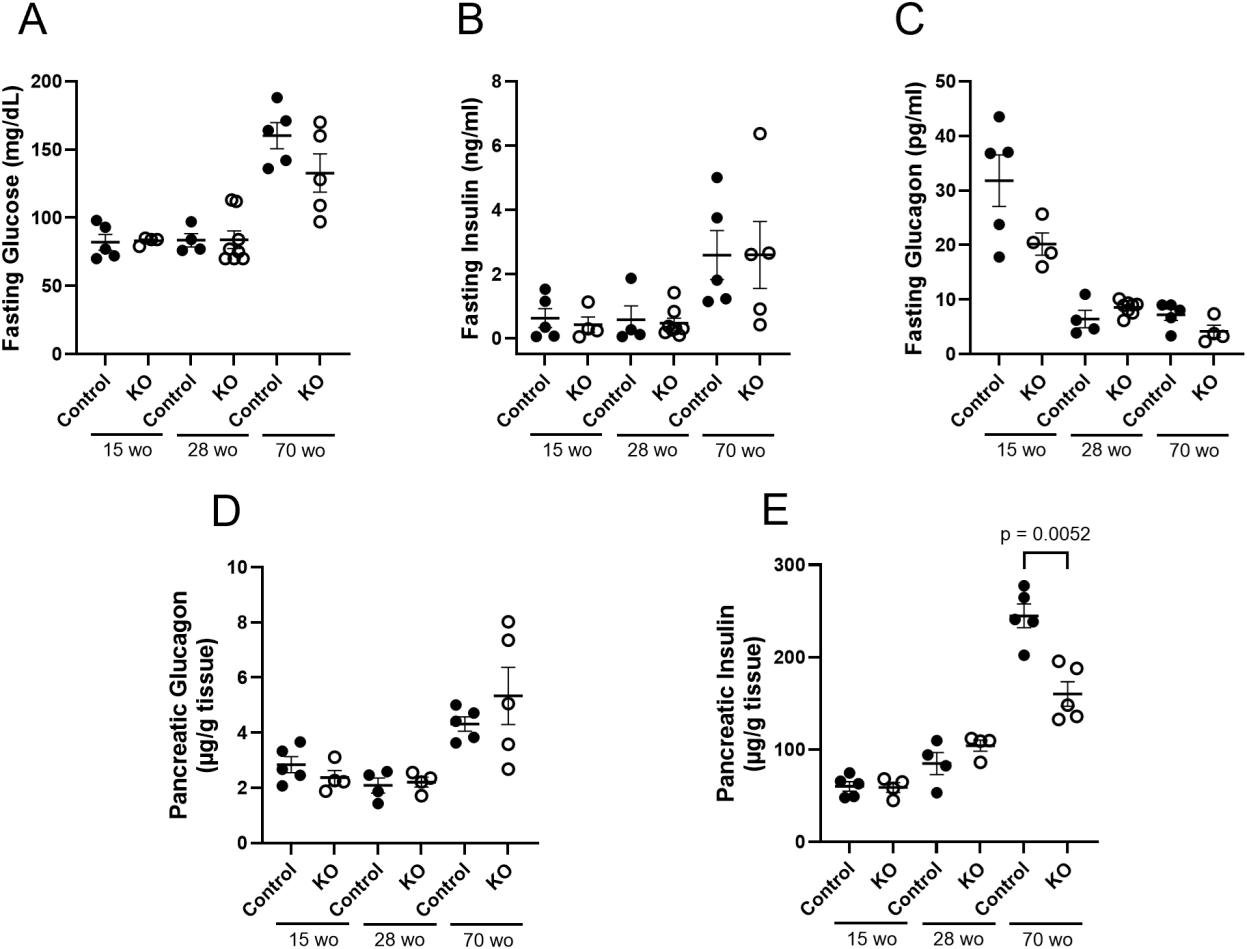
Control and β-cell OXTR knockout (KO) mice maintained on a LFD and at the indicated age (week old; wo) fasting blood samples were measured for glucose **(A)**, insulin **(B)**) and glucagon **(C)** levels. Pancreata were collected at the same time points for measurement of total tissue glucagon **(D)** and insulin **(E)** expressed as µg of hormone per g of pancreas weight. Each point represents one mouse and data are expressed as mean ± SEM.

In order to examine plasma and pancreatic hormone levels over the course of time, samples were analyzed from LFD control and β-KO mice at 15, 28 and 70 weeks of age (**Figure 8**). There were no group differences over time in fasting plasma glucose, insulin or glucagon (**Figures 8A–C**). While there were no differences in pancreatic glucagon content at any time point (**Figure 8D**), there was a significant reduction in pancreatic insulin in β-KO mice relative to control at 70 weeks of age (**Figure 8E**).

### 3.5 Islet Histopathology

**Figure 9:**
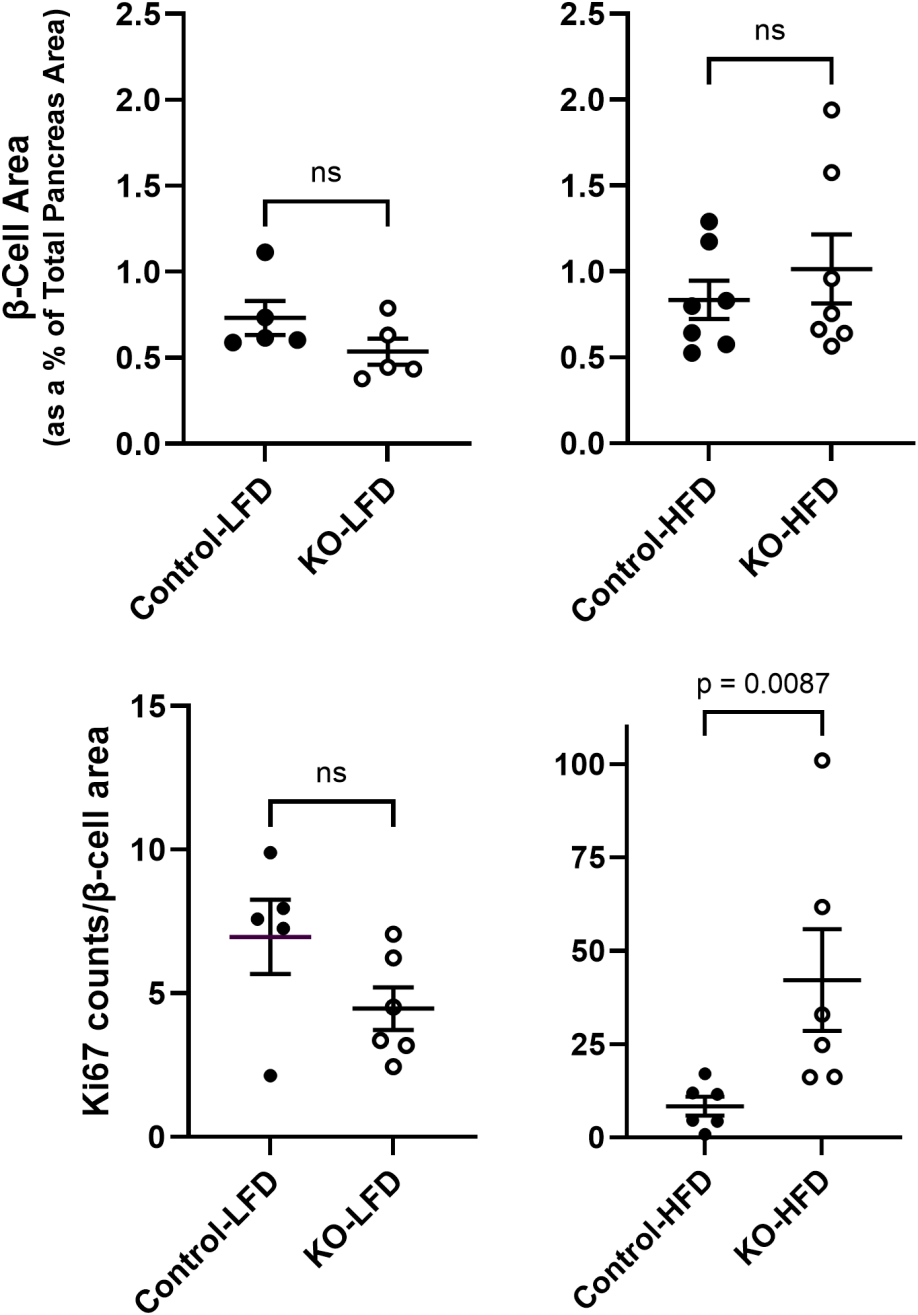
Pancreatic β-cell area and Ki-67 expression from control and β-cell OXTR knockout (KO) mice that were maintained on a low-fat diet (LFD) or high-fat diet (HFD) over a 23-week study period. β-cell area was estimated by insulin positive immunoreactive area expressed as a percent of the total pancreas area **(A)**. Ki-67 immunoreactive cell nuclei were counted in the same tissue sections and normalized for total β-cell immunoreactive area **(B)**. Each data point is from one mouse and represents the mean value from 5 tissue sections separated by 100 µm for each mouse. Data are expressed as the mean ± SEM and significance indicated above the bar in each figure.

It is well established that HFD leading to hyperglycemia and hyperlipidemia results in metabolic stress that initiates changes in islet cell composition and function (20, 21). Therefore, β-cell mass (estimated as the insulin immunoreactive area relative to the total pancreatic area) was measured in the LFD and HFD mice at study endpoint. There were no significant differences in β-cell mass between control and β-KO mice in either the LFD or HFD groups (**Figure 9A**). We also evaluated measures of apoptosis and cell proliferation using the terminal deoxynucleotidyl transferase dot nick end labeling (TUNEL) method and immunodetection of Ki-67, respectively. TUNEL positive staining was rare (on average < 1 TUNEL positive cell per islet) in the β-cells and was not different between control and β-KO mice for either dietary group (data not shown). In contrast, there was a significant increase (p = 0.009) in Ki-67 positive cells in the β-KO-HFD group compared to the control-HFD group (**Figure 9B**). There was no difference in Ki-67 expression between control and β-KO mice in the LFD group. Taken together, these data suggest that while deletion of the β-cell OXTR is not associated with loss of β-cell mass and apoptosis, it appears to stimulate cellular proliferation in the HFD condition.

### 3.6 Role of OXTR in cytoprotection

**Figure 10:**
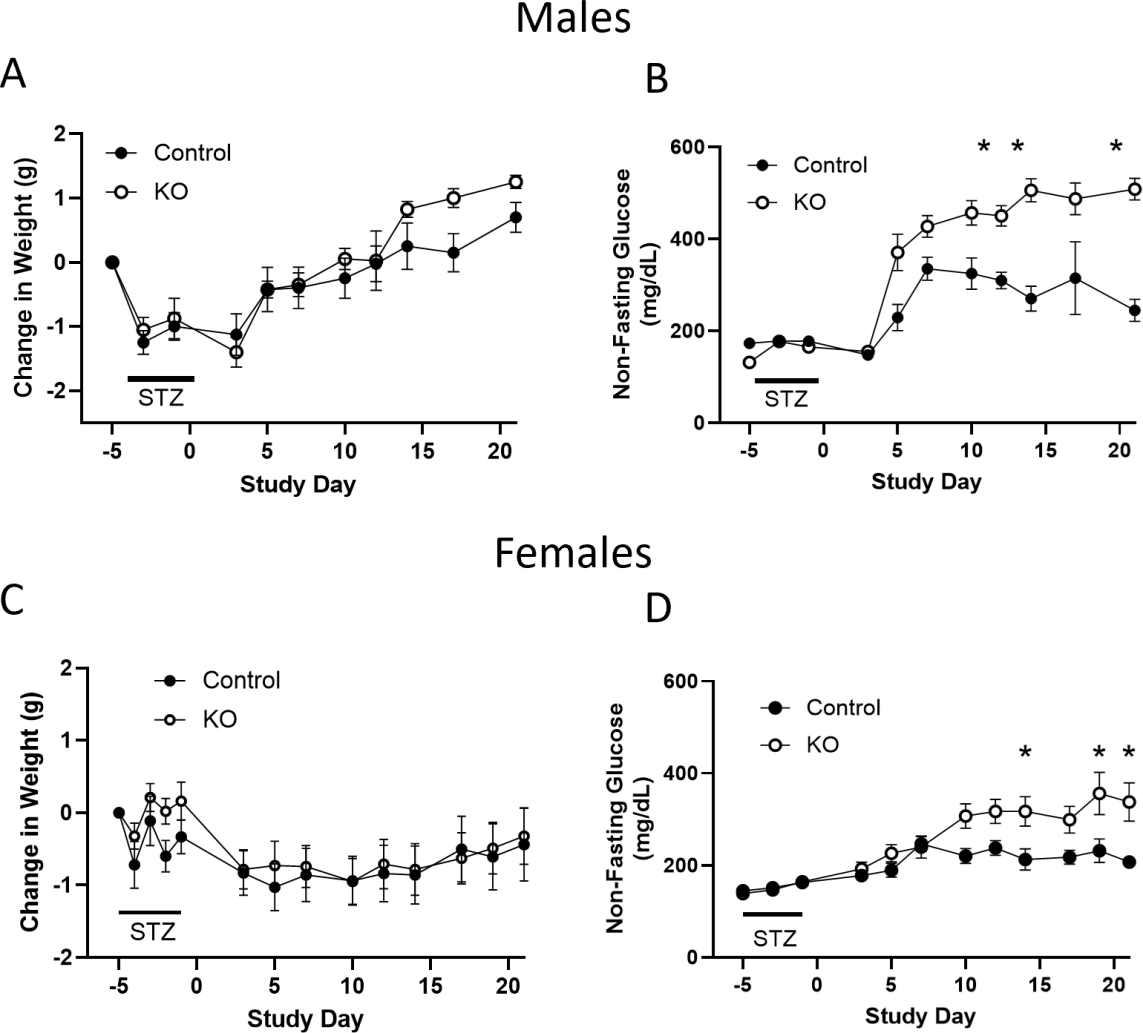
Control and β-cell OXTR knockout (KO) male mice were treated with low dose (50 mg/kg) streptozotocin (STZ) on four consecutive days and changes in body weight **(A)** and non-fasting blood glucose **(B)** were monitored over 21 days. **(C, D)**, weight and non-fasting glucose for female mice, respectively. Data are expressed as the mean ± SEM; n=10 mice per group. The change in glucose over time was significantly different in β-KO compared to control mice by repeated measures ANOVA (p = 0.0023 for males and p = 0.029 for females) and significant differences at each time point indicated by * = p < 0.05). There was no significant difference in weight change between groups for males or female mice.

There is evidence that the OXTR in β-cells may play a role in cytoprotection following metabolic stress (22, 36). To examine this possibility, β-KO and control mice were given low dose STZ (an alkylating agent that is toxic to β-cells) injections for 4 consecutive days, and blood glucose levels were monitored as an index of β-cell loss. There were no significant group differences in body weight change (**Figure 10A**), however, blood glucose levels were significantly higher in β- KO mice relative control mice over the course of the study (**Figure 10B**). It has been shown that female mice are more resistant to STZ than male mice (37), therefore, we repeated the study in a cohort of female control and β-KO mice. As was the case for males, there were no significant differences in body weight change between groups in females (**Figure 10C**), and glucose levels were significantly higher in the β-KO mice than the control group (**Figure 10D**). These data suggest that OXTR may provide significant β-cell protection in both male and female mice in the face of STZ-mediated cytotoxicity.

## 4 Discussion

The current study provides further evidence of an intrinsic OXT/OXTR network within the pancreatic islet. Previous reports have established that OXTR protein is expressed in β- cells and α-cells (22, 24, 38), with greater expression in α-cells relative to β-cells. Given that commercially available antibodies for OXTR have poor reliability and validity (39), in the current study *in situ* mRNA hybridization was used to detect OXTR expression in islets. We identified OXTR expression in β-, α- and δ-cells of the mouse islet. These data are consistent with publicly available mouse islet single-cell RNAseq data demonstrating the highest expression of OXTR in α-cells, followed by δ-cells, then β-cells (40, 41). Our data revealed that in the β-cell OXTR-KO mice, RT-qPCR of whole islets showed a 40% reduction in OXTR message relative to control mice and, we interpret this decrease to be specific to loss of expression within β-cells and further suggest that the majority of OXTR is present in α- and δ-cells. However, in the context of a HFD, which is characterized by increased inflammation (20, 21), β-cell OXTR mRNA expression was increased ~5-fold in control mice compared to LFD mice. Previously, it has been demonstrated in various models of inflammation and in different tissues that OXTR expression is dramatically upregulated (42–50), suggesting this may be a fundamental aspect of this receptor’s regulation. In contrast to the widespread islet OXTR expression, we found that OXT expression was confined to the β-cells, which is consistent with previous reports (23, 25). It is tempting to speculate that local β-cell OXT secretion works in an autocrine or paracrine manner to regulate OXTR function within the islet.

In order to manipulate this local OXT/OXTR system, we selectively knocked out the OXTR gene from pancreatic β-cells. The β-KO was verified by genotyping, RT-qPCR mRNA expression levels, and *in situ* hybridization of cellular mRNA. We further confirmed the knockout functionally in islets harvested from β-KO mice, in which the ability of OXT to stimulate insulin secretion under high glucose conditions was abolished compared to control islets. In contrast, OXT had no effect on insulin secretion under low glucose conditions, suggesting a context (hyperglycemia) specific effect of OXT on insulin section. Glucagon secretion was stimulated in islets from both control and β-KO mice under low glucose conditions likely through activation of OXTRs in α-cells, demonstrating preservation of α-cell OXTR function. Whereas *in vivo* administration of OXT to hyperglycemic control mice reduced blood glucose, it had no effect in the β-KO mice. It is worth noting that the OXT-mediated decrease in blood glucose was only achieved in the control mice given supraphysiological doses of OXT (1mg/kg i.p.) that increased plasma levels of OXT to greater than 5,000 pg/ml (compared to OXT levels in untreated mice of ~60-80pg/ml (48)). Notwithstanding, our data show that the loss of the OXT response was specific to deletion of the β-cell OXTR, and not due to OXTR receptors in other tissues or cell types.

The *in vitro* data described above suggest that OXT acts as an incretin for insulin secretion from β-cells during hyperglycemia, and as an incretin for glucagon from α-cells during hypoglycemia. The OXTR is a G protein coupled receptor whose structure and function have been extensively reviewed (51–53). When stimulated by OXT, OXTR activates the Gq/11 signaling pathway, which ultimately leads to phosphorylation of PKC. It has been shown that activation of PKC amplifies secretion of insulin (54) and glucagon (55). This OXTR enhancement of insulin secretion only occurs in the presence of elevated glucose (i.e., GTT) whereas the amplification of glucagon secretion only occurs when glucose is low. Therefore, OXTR’s effect is an amplification of secretion that is triggered by a separate glucose dependent mechanism. It is proposed that the mechanism by which the OXTR- Gq/11-PKC pathway amplifies hormone secretion is by directly influencing “release competency” of insulin or glucagon containing vesicles (54).

The role of β-cell OXTR in the regulation of glucose homeostasis was assessed in the β-KO mice subjected to LFD or HFD conditions. Despite the data showing the *in vitro* loss of β-cell OXTR function mentioned previously, deletion of β-cell OXTR had no detectable effects on blood glucose, body weight gain or measures of insulin sensitivity in either LFD or HFD conditions (23 weeks on diet). Therefore, in contrast to the acute glycemic effects seen with islets *in vitro* and in the pharmacological studies *in vivo*, β-cell KO of the OXTR did not impair glucose tolerance in mice on LFD or HFD. It is possible that there are adequate compensatory mechanisms able to overcome the loss of any OXTR/OXT incretin-like activity under the experimental conditions studied such that differences in glycemic control could not be detected. Since our data do not support a role for β-cell OXTR in whole body glycemic regulation, it appears that the metabolic changes seen in the global OXT and OXTR knockout studies may be due to extra-pancreatic mechanisms.

It is well established that chronic hyperglycemia and hyperlipidemia can lead to β-cell dysfunction and pathophysiological changes leading to type 2 diabetes (20, 21). It has also been suggested that the islet OXT/OXTR system may play a role in the attenuation of these pathological processes (22, 23, 36). In the present study, deletion of the β-cell OXTR did not significantly affect β-cell mass or measures of apoptosis following a HFD. Although these results differ from previously published work, there are methodological differences among the studies. Our studies were based on data from histological evaluation of pancreatic tissues collected from control and β-KO mice after diet manipulation at study endpoint, while studies by others evaluated cell death in isolated islets exposed to cytotoxic agents (22) or *in vitro* viability of islets or β-cell lines that were exposed to metabolic stressors (23). Although we did not find changes in β-cell mass or apoptosis in the β-KO mice, we did observe reduced insulin content in whole pancreas extracts of aged mice compared to controls. Additionally, a measure of cell proliferation was significantly increased in β-cell KO mice relative to control on a HFD. We speculate that the increase in cell proliferation may be a compensatory response to β-cell exhaustion needed to maintain glucose regulation in the face of reduced β-cell insulin content in the β- KO mice.

In order to investigate cellular protective effects mediated by the OXTR we exposed control and KO mice to low dose STZ treatment. Whereas a long-term HFD can lead to gradual changes in β-cell health, STZ treatment results in acute β-cell cytotoxicity. It was found that β-KO led to higher glucose levels than in control mice, suggesting that the β-cell OXTR provides some cellular protection against STZ-mediated cytotoxicity.

In conclusion, the present study provides further evidence for an intrinsic OXT/OXTR network in the pancreatic islet. We generated and validated a β-cell-specific OXTR knockout mouse in order to investigate the role of this local system in glycemic regulation and islet health. *In vitro* experiments and *in vivo* pharmacologic experiments provided evidence that under hyperglycemia, OXTR activation can potentiate insulin secretion and glucose suppression. On the other hand, loss of the β-cell OXTR had no significant effect on glucose tolerance in either LFD or HFD conditions, suggesting that the β-cell OXTR did not directly affect whole body glycemic regulation in the experimental models used in this study. This does not preclude the possibility that OXTR in α-cells or δ-cells may participate in glycemic regulation. We also provided evidence that the β-KO reduces pancreatic β-cell insulin content and stimulates islet β-cell proliferation. Finally, β-cell OXTR protects against STZ-induced hyperglycemia, presumably by attenuating cytotoxicity. This is consistent with the well-established role of OXT/OXTR in attenuating inflammation and oxidation.

## Data Availability

The raw data supporting the conclusions of this article will be made available by the authors, without undue reservation.
