# Supplementary material for: Targeted deletion of the pancreatic β-cell oxytocin receptor and its effects on metabolic regulation and β-cell health
Source: Front Endocrinol (Lausanne). 2024 Dec 23;15:1465818. doi: 10.3389/fendo.2024.1465818 (PMC11700818; doi:10.3389/fendo.2024.1465818)
Supplement: Supplementary file 1 [file DataSheet1.pdf]

Supplementary Material

Supplementary Table 1. mRNA Expression from Control and OXTR β-Knockout Mice Islets

Gene	Control	OXTR β- KO	p
OXTR	1.001 ± 0.001	0.603 ± 0.083	0.009
OXT	1.001 ± 0.001	1.431 ± 0.288	0.210
AVPR1a	1.004 ± 0.004	0.681 ± 0.212	0.203
AVPR1b	1.001 ± 0.001	0.742 ± 0.163	0.189
INS2	1.001 ± 0.001	1.582 ± 0.430	0.248
GCG	1.000 ± 0.000	0.793 ± 0.114	0.144
PDX1	1.002 ± 0.002	1.000 ± 0.253	0.993

Control and β-knockout (KO) islets were isolated and relative mRNA expression was determined by RT-qPCR. Data was normalized to expression in WT islets. Data represents mean ± SEM (n= 3 islet isolations per group, each isolation represented islets pooled from 5 male mice).

Supplementary Table 2. Tissue OXTR mRNA Expression in control and OXTR β-Knockout Mice

Tissues	Control	OXTR β- KO	p
Brain	1.024 ± 0.106	0.915 ± 0.072	0.419
Heart	1.058 ± 0.158	0.778 ± 0.137	0.219
Epdidymal Fat	1.026 ± 0.125	1.382 ± 0.204	0.175

Control and β-knockout (KO) islets were isolated and relative mRNA expression was determined by RT-qPCR. Data was normalized to expression in WT islets. Data represents mean ± SEM; n = 5.

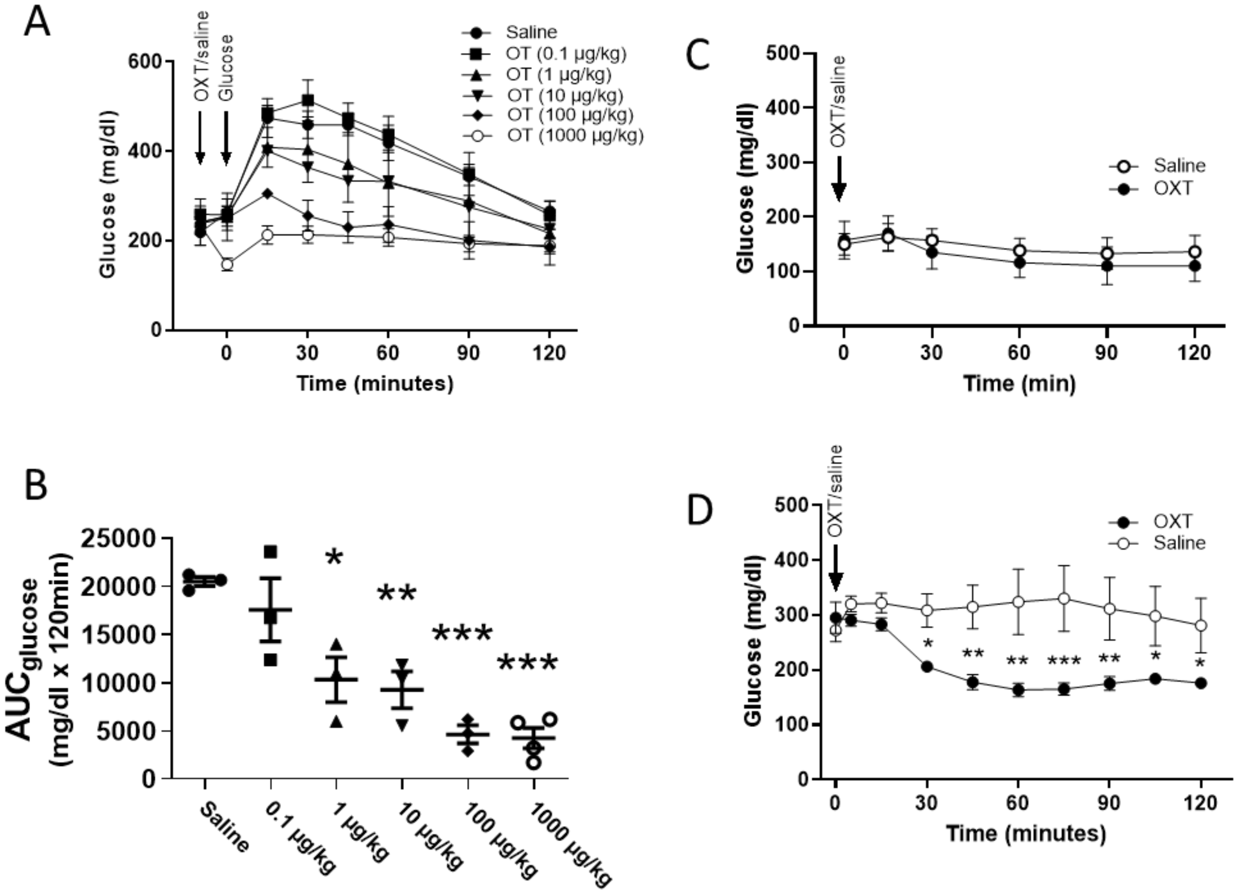

Supplementary Figure 1. Effect of exogenous oxytocin (OXT) administration on blood glucose in mice. Panel A. C57B/6 mice fasted for 6 hours were given i.p. injections of OXT at the indicated doses followed by an ipGTT, n= 3 mice per dose. Panel B. The area under the curve for glucose ($AUC_{glucose}$) for data from Panel A. Panel C and D. Saline or 1 mg/ml OXT was injected i.p. into normoglycemic control mice maintained on a normal chow diet (C) or in mice fed a high fat diet for 12 weeks to induce hyperglycemia (D) and blood glucose levels were monitored over 2 hours, n= 10 mice per group. Mice were between 20 and 24 weeks old)All data are the mean ± SEM; *, **, *** indicates $p < 0.05$, <0.01, <0.001, respectively.

Reagents

Item	Company	Catalog #	Notes
Teklad global 18% protein (low-fat diet)	Inotiv, Inc	2918	
Rodent Diet With 60 kcal% Fat	Research Diets	D12492i	
Streptozotocin	Cayman Chemical	13104	50 mg/kg
Dextrose Solution 50% (Glucose)	Phoemix Pharmaceuticals	10771	2 g/kg
Insulin (Humulin R U-100)	Eli Lilly	00002821501	0.8 U/kg
Oxytocin	Bachem	4016373	1000 µg/kg
Insulin ELISA	Mercodia	10-1247-01	
Glucagon ELISA	Mercodia	10-1281-01	
T-PER™ Tissue Protein Extraction Reagent	Thermo Fisher	78510	
Millicell Cell Culture Insert, 12 mm, polycarbonate, 3.0 µm	Millipore	PITP01250	
TUNEL Assay	Roche	12156792910	
**qRT-PCR for mRNA Expression**			
18S primer	Thermo Fisher	Mm03928990_g1	
OXTR primer	Thermo Fisher	Mm01182684_m1	
AVPR1a primer	Thermo Fisher	Mm00444092_m1	
AVPR1b primer	Thermo Fisher	Mm01700416_m1	
INS2 primer	Thermo Fisher	Mm07293269_g1	
GCG primer	Thermo Fisher	Mm00801714_m1	
OXT primer	Thermo Fisher	Mm01329577_g1	
PDX1 primer	Thermo Fisher	Mm00435565_m1	
RNeasy Mini Kit	Qiagen	74106	
Qiazol	Qiagen	79306	
High Capacity cDNA Reverse Transcription Kit with RNase Inhibitor	Applied Biosystems (Thermo Fisher)	4374966	
TaqMan Fast Universal PCR Master Mix	Applied Biosystems (Thermo Fisher)	4366072	
**Immunohistochemistry Antibodies**			
Insulin antibody	DAKO	A0564	1:2000

Glucagon antibody	Santa Cruz Biotechnology	sc-514592	1:100
Oxytocin antibody	Proteintech	18041-1-AP	1:100
Somatostatin antibody	Thermo Fisher	MA5-16987	1:100
Ki67	Thermo Fisher	14-5698-80	1:100
Donkey Anti-Guinea Pig Alexa Fluor 488	Jackson ImmunoResearch	706-545-148	1:500
Donkey Anti-Rat IgG Alexa Fluor 594	Jackson ImmunoResearch	712-585-153	1:500
Donkey Anti-Mouse IgG Alexa Fluor 594	Jackson ImmunoResearch	715-585-150	1:500
Donkey Anti-Mouse IgG Alexa Fluor 647	Jackson ImmunoResearch	715-605-150	1:500
Donkey Anti-Rabbit IgG Alexa Fluor 647	Jackson ImmunoResearch	711-605-152	1:500
Donkey Anti-Rabbit IgG Alexa Fluor 790	Jackson ImmunoResearch	711-655-152	1:500
***in situ* mRNA Hybridization**			
RNAscope™ Probe- Mm-Oxtr-O2-C3	Advanced Cell Diagnostics	901851-C3	GenBank accession number NM_001081147.2, target region 30 – 884
RNAscope™ Probe- Mm-Oxtr	Advanced Cell Diagnostics	412171	GenBank accession number NM_001081147.1, target region 776 - 1676
RNAscope™ Probe- Mm-Ins2-O1-C2	Advanced Cell Diagnostics	497811-C2	GenBank accession number NM_008387.5, target region 2 - 470
RNAscope™ Probe- Mm-Gcg-O1	Advanced Cell Diagnostics	482311	GenBank accession number NM_008100.4, target region 2 - 1073
RNAscope™ Probe- Mm-Sst-C2	Advanced Cell Diagnostics	404631-C2	GenBank accession number NM_009215.1, target region 18 - 407

RNAscope™ Probe- Mm-Oxt	Advanced Cell Diagnostics	493171	GenBank accession number NM_011025.4, target region 7 - 473
Perkin Elmer Reagent Kit, 4-color kit	Akoya Biosciences	NEL794001KT	
